# Effect of Breastfeeding on Serum Osteoprotegerin and Soluble Receptor Activator of Nuclear Factor-Kappa B Ligand in Full Term Neonates

**DOI:** 10.5812/ircmj.7591

**Published:** 2013-10-05

**Authors:** Mandana Rafeey, Amir Ghorbanihaghjo, Fardad Masoumi, Samira Alizadeh, Sina Davari Farid

**Affiliations:** 1 Liver and Gastrointestinal Disease Research Center, Tabriz University of Medical Sciences, Tabriz, IR Iran; 2 Biotechnology Research Center, Tabriz University of Medical Sciences, Tabriz, IR Iran; 3 Shams General Hospital, Tabriz, IR Iran

**Keywords:** Breast Feeding, Infant Formula, Osteoprotegerin, sRANKL, Tumor Necrosis Factor-Alpha (TNFα)

## Abstract

**Background:**

Human breast milk, the sole source of nutrition during the early neonatal period, is rich in nutrients, hormones, growth factors, and immunoactive molecules, which influence the growth, development, and immune status of the newborn infant. It had long been thought that breast milk is an adequate source of anthracitic activity for the newborns and growing child.

**Objective:**

Human milk is a complex biologic fluid which contains nutritional and protective factors such as Osteoprotegerin (OPG), at levels 1000-fold higher than normal human serum. Since OPG and Receptor activator of nuclear factor-kappa B ligand (RANKL) system are tightly involved in bone remodeling and immune activity, the study was designated to evaluate the effect of breastfeeding on serum soluble receptor activator of nuclear factor-kappa B ligand (sRANKL) /OPG ratio in full term neonates in comparison with those of formula feeding full term neonates.

**Materials and Methods:**

In this cross-sectional study serum levels of OPG and sRANKL in 45 breastfed infants were compared to those of 44 formula-fed full term infants. The levels of serum OPG, sRANKL, and Tumor necrosis factor alpha (TNFα) were determined by standard techniques using enzyme-linked immunosorbent assay kits.

**Results:**

The serum levels of OPG were significantly higher (P < 0.001), and the concentrations of TNFα was markedly lower (P = 0.024) in breastfed infants than those of formula-fed infants. No marked differences were observed between the serum levels of sRANKL in the two study groups (P = 0.8).

**Conclusions:**

High OPG and low TNFα levels in serum of breastfed infants are important factors involved in remodeling of bone, and immune activity may prove superiority of breastfeeding over formula feeding during infancy.

## 1. Background

Human breast milk, the sole source of nutrition during the early neonatal period, is rich in nutrients, hormones, growth factors, and immunoactive molecules, which influence the growth, development, and immune status of the newborn infant (1). It had long been thought that breast milk is an adequate source of anthracitic activity for the newborns and growing child (2). Given the importance of breastfeeding in preventing diseases, and many benefits for mother and infant, termination of breastfeeding especially in a first year of life would be harmful for mothers, children and society (3). The childhood is most important period for the processes governing normal bone remodeling and the development of immune responses. During childhood, bone formation exceeds resorption and thereby ensures that osteogenesis parallels the rapid changes in size and weight during infancy (4). Bone remodeling is tightly regulated by a molecular triad composed of osteoprotegerin (OPG)/Receptor activator of nuclear factor-kappa B ligand (RANKL)/receptor activator of nuclear factor B (RANK) (5, 6). RANKL is a key mediator of bone resorption (7, 8), which is expressed as membrane-bound or soluble forms (sRANKL) by tissues as diverse as lymph nodes, spleen, thymus, and bone-forming cells. sRANKL by binding to its cognate receptor, RANK, on osteoclast and their precursors stimulates osteoclastogenesis (9). By contrast, OPG is the main inhibitor of bone resorption because of its function as a soluble decoy receptor for sRANKL (6). Therefore, the equilibrium between OPG and sRANKL in the bone microenvironment regulates bone resorption (6). OPG is widely expressed in multiple tissues, including lung, heart, kidney, and placenta (10), as well as in mammary gland epithelial cells (11).It has been shown that human breast milk contains a substantial amount of OPG (12). The active OPG is present in human milk samples at different times during lactation in concentrations that are up to 1000-fold higher than that found in normal human serum. Furthermore, it is reported that human breast milk cells and the human mammary epithelial cell line MCF-7 express OPG. Also, *In vitro* studies demonstrated that milk OPG is bioactive and suggested that it may contribute to the antiresorptive activity of milk on bone. If, as suggested by the preliminary experiment showed that human milk-derived OPG administered orally to rat pups may be absorbed across the gastrointestinal tract to reach the systemic circulation and so, it is tempting to speculate that it may modulate sRANKL/RANK interactions, not only in the intestine lumen but also in underlying tissues (4).

## 2. Objectives

Since breast and formula milks are the sole sources of nutrition, growth factors, and other factors for infants after birth and due to the presence of active OPG in human milk and its role in bone homeostasis, the serum levels of OPG and sRANKL were measured in breast feeding infants and formula feeding and results were compared to each other.

## 3. Materials and Methods

### 3.1. Participants

This cross-sectional study was performed in Tabriz University of Medical Sciences, between June 2008 and January 2009. The research project was reviewed and approved by the Ethics Committee of Tabriz University of Medical Sciences. The study groups were composed of 89 normal healthy newborns aged 20 to 100 days. Forty five neonates were solely fed with breast milk since birth (30 male and 15 female) as the case group (Group A) and 44 neonates were exclusively fed with formula milk, as the control group (27 male and 17 female) (Group B). Data regarding age, sex and weight, type of feeding (breast or formula feeding), and a detailed medical history were recorded. Newborns were included, according to the following criteria: gestational age between 37 and 42 weeks (full term newborns) (13), absolute feeding with breast milk or formula milk, and whose mother had given consent after being adequately informed. Exclusion criteria were newborns with septicemia, severe anemia, need for neonatal intensive care unit (NICU), hemolytic disease, infection, experience of getting Fresh frozen plasma (FFP) and other blood products, and any disease or use of medication known to affect bone metabolism, we also excluded newborns whose mothers were smokers or drug abusers. Venous blood samples were obtained from the infants and the sera were separated immediately and analyzed for calcium (Ca), phosphorous (P), and alkaline phosphates (ALP). The remaining serums samples were stored at -70°C until further analysis.

### 3.2. Analytical methods

Serum calcium, phosphorous and alkaline phosphates were analyzed using an automated chemical analyzer (Abbott Analyzer, Abbott Laboratories, Abbott Park, North Chicago, IL). Serum OPG, TNFα and sRANKL concentrations were determined by commercially Enzyme-Linked Immunosorbent Assay (ELISA) kits; sRANKL (total) by Bio Vendor kit (Lot. No: RD-1839), OPG and TNFα by Bender Med Systems GmbH (Vienna, Austria, Lot. No: 36437010 and 11096013, respectively).

### 3.3. Statistical analysis

Results were expressed as means ± standard deviation, or median as appropriate. The Kolmogorov– Smirnov test was used to evaluate the distributions, and Independent T-test and Mann–Whitney U tests, as appropriate, were used to assess the significance of the differences between the two groups. The correlations were evaluated using the Spearman's test, and P < 0.05 was considered statistically significant. Statistical analyses were performed using SPSS software package version 13 for Windows (SPSS Ins, Chicago, IL).

## 4. Results

Table 1 summarizes the demographic data and laboratory findings from the breastfeeding and formula feeding groups. 

**Table 1.** Summarizes the Demographic Data and Laboratory Findings From the Breastfeeding and Formula Feeding Groups

Variable	Breast Feeding Group (n = 45)	Formula Feeding Group (n = 44)	P value ^a, b^
**Age (Days) ^c^**	37.44 ± 12.16	42.80 ± 13.82	0.056
**Weight (g) ** ^**c**^	3681.39 ± 855.63	3598.64 ± 798.66	0.850
**Gender (Male/Female)**	30/15	27/17	0.602
**Serum Ca** ^**2+**^ **** ^**c , d**^ ** (mmol/L) ** ^**e**^	1.17	1.15	0.895
**Serum P ** ^**d**^ ** (mg/dL) ** ^**c**^	5.70 ± 1.50	6.02 ± 1.34	0.283
**Serum ALP ** ^**d**^ ** (IU/L) ** ^**c**^	986.56 ± 435.55	863.55 ± 386.43	0.163

^a^ Breast feeding group vs. formula feeding group

^b^ P < 0.05 is significant

^c^ Mean ± SD

^d^ ALP, Alkaline phosphates; Ca, Calcium; P, Phosphor

^e^Median

**Figure 1.**
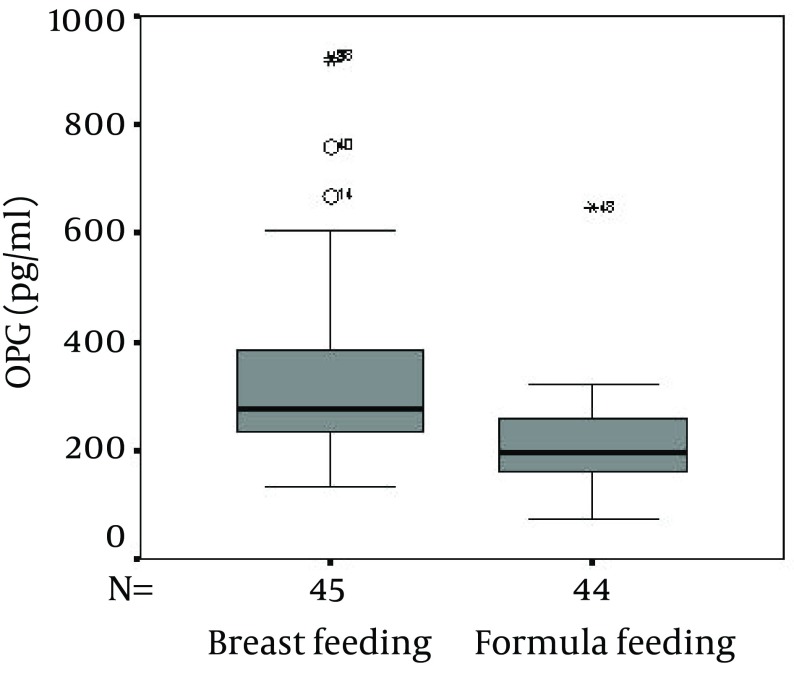
Comparison Between the Osteoprotegerin (OPG) Concentrations in the Breast and Formula Feeding Groups (P = 0.001)

**Figure 2.**
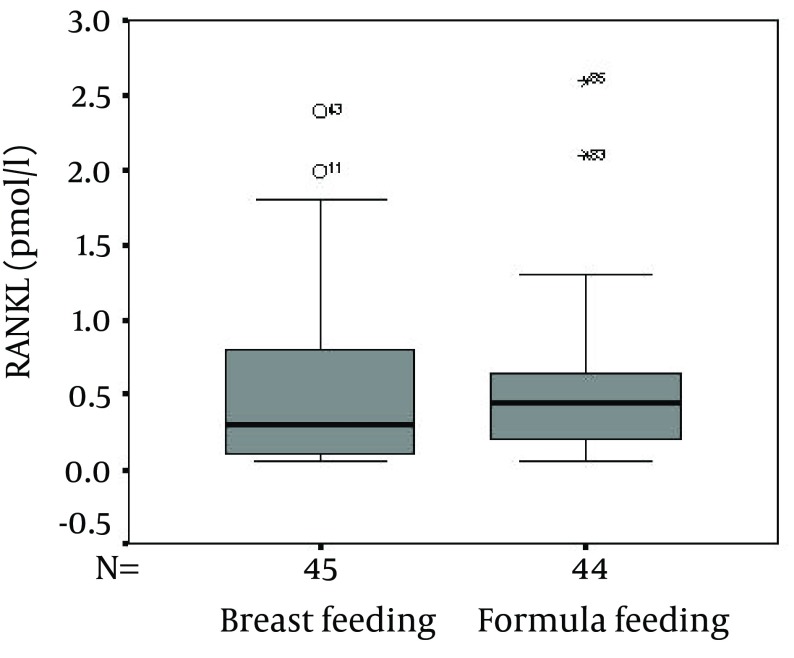
Comparison Between the Receptor Activator of Nuclear Factor B Ligand (RANKL) Concentrations in the Breast and Formula Feeding Groups (P = 0.7)

No significant difference was present between the two groups regarding age, weight, gender, and also serum Ca, P, ALP and sRANKL. As shown in the Figure 1, although the serum OPG concentration was significantly higher in the breastfeeding than that of the formula feeding (P = 0.001), no significant difference was found between the sRANKL levels in the breast and formula feeding groups (P = 0.7) (Figure 2). 

The serum ratio of OPG/sRANKL decreased also in neonates fed with breast, but it was not statistically significant (P = 0.057). The serum TNFα levels were markedly lower in breast-fed infants compared to formula-fed ones (P = 0.024).

## 5. Discussion

In this study for the first time the concentrations of OPG, sRANKL, and TNFα the biomarkers of inflammation and bone homeostasis were simultaneously determined and compared in breast feeding and formula feeding infants. It is widely accepted the diverse and compelling advantages of breastfeeding due to not only the value of different nutrients, but also the availability of the bioactive proteins (14). Breast milk composition is therefore the best food source during the early neonatal period (1). OPG is one of the active compounds of milk (4). OPG or osteoclast oogenesis inhibiting factor (OCIF), a member of the TNF superfamily, inhibits maturation of osteoclasts through binding to sRANKL (15). Bone remodeling appears to be mainly controlled by the balance in OPG/sRANKL ratio. Every modification in the OPG-to-sRANKL ratio can induce either an excessive bone resorption or, in contrast, an excessive bone formation (16, 17). Also OPG decreases the production of cytokines in response to dendritic cell stimulation by sRANKL, i.e., proinflammatory cytokines such as interleukin-6 (IL-6), IL-11, and also decreases the production of cytokines (IL-12 and IL-15) by proliferating T cells, therefore OPG may also be pivotal in modulating the immune system (18). The main finding of our study was higher levels of OPG in breastfed infants, compared to newborns fed with formula. Although we observed differences in OPG/sRANKL ratio between the two groups (higher OPG/sRANKL ratio in breast-fed), but it was not statically meaningful.Known inducers of bone resorption and hypocalcaemia, such as IL1 and TNFα, act indirectly through the production of RANKL (19). TNFα is the key polypeptide mediator synthesized in response to injury and infectious, inflammatory, and immunologic challenges (20).In our study, breast fed infants presented lower serum TNFα concentrations than did formula fed infants. These results differ from others (20, 21), who detected no differences in serum TNFα concentrations between breast- and formula-fed infants.

Granot E et al. mentioned that breast-fed and formula-fed infants differ in the amount and type of polyunsaturated fatty acids consumed. The fatty acid composition of cell membranes is related to dietary fatty acids and, in adults, changes in membrane fatty acid composition are accompanied by changes in monocyte cytokine production, and hence a modification of the immunologic response. They reported that the release of proinflammatory cytokines such as TNFα by immunocompetent cells does not differ significantly in breastfed and formula-fed infants, despite differences in cell membrane fatty acid composition (20). Although the levels of serum Ca^2+^, P concentrations and the activity of ALP in the present study confirmed the finding of the others, some investigator reported that there are no statistically significant differences among breast and formula feeding infants in serum Ca, P, and ALP at birth, and also serum Ca and ALP were not different in the two groups at 6 and 12 months of ages (21, 22).Further studies with a larger sample size are required to confirm these findings. Meanwhile, to best understand the effect of OPG in breast milk On neonatal period, the correlation between OPG level of breast milk and other biochemical markers of bone remodeling, such as serum C-telopeptide of type 1 collagen, urine N-telopeptide of type 1 collagen, precollege type I extension peptides, and osteocalcin must be further studied.The main findings of this cross-sectional study were higher levels of OPG as an antiresorptive agent, and lower TNFα concentrations as an inflammatory marker among the breastfed neonates, compared to formula fed ones.
